# Huashan perioperative nursing program for stroke patients undergoing contralateral seventh cervical nerve transfer

**DOI:** 10.1186/s13741-022-00245-4

**Published:** 2022-04-07

**Authors:** Fan Su, Ye Xu, Xiaoqian Wang, Yiqun Zhou, Wendong Xu, Yaojin Zhang, Ying Liu

**Affiliations:** 1 Department of Hand and Upper Extremity Surgery, Jing’an District Central Hospital, Fudan University, No. 259 Xikang Rd., Shanghai, 200040 China; 2 Department of Hand Surgery, Huashan Hospital, Fudan University, No. 12 Middle Wulumuqi Rd., Shanghai, 200040 China

**Keywords:** Stroke, Contralateral seventh cervical nerve transfer, Evaluation and education, Postoperative monitoring, Targeted nursing

## Abstract

**Background:**

A previous investigation regarding contralateral seventh cervical nerve transfer (CC7) revealed a novel and effective approach to improve arm function in patients with chronic spastic paralysis. The patients who underwent both CC7 and standard rehabilitation showed greater functional improvements and spasticity reductions than patients in the control group, who underwent rehabilitation only. Additional efforts are needed to maximize the benefits in patients and establishing a supporting nursing program is a promising method for achieving this goal.

**Methods:**

The present Huashan nursing program was established in consideration of the following elements: providing routine perioperative care, ensuring surgical safety, and improving patient cooperation. Before surgery, psychiatric nursing, health education, and risk control were emphasized. After surgery, in addition to routine nursing and positioning, special attention was needed for targeted nursing in cases of postoperative adverse events. In addition, we performed descriptive statistical analysis of the clinical data of patients participating in the Huashan nursing program, focusing on postoperative adverse events. In total, 85 patients were included in the study, 10 of whom experienced adverse events, including severe pain (5, 5.88%), neck hematoma (2, 2.35%), dyspnea (2, 2.35%), and hoarseness (1, 1.18%). The above adverse events were alleviated through the targeted nursing care guided by the Huashan program.

**Discussion:**

This article introduces the Huashan nursing program, which is based on preoperative evaluations, educational sessions, postoperative monitoring, and targeted nursing, for patients undergoing CC7. This nursing program helped promote and provided the opportunity to maximize the benefits of CC7.

## Background

Stroke causes various types of functional impairments, which persist years following stroke onset and may even be permanent. Despite advances in medicine, the overall stroke burden has been reported to continuously increase in recent years (Feigin et al., 2017; Feigin et al., 2014). Urgent demands for stroke recovery have been proposed, and a study on contralateral seventh cervical nerve transfer (CC7) provided new insights into the treatment of chronic stroke. By transferring the C7 nerve, a functional connection between the spastic arm and ipsilateral hemisphere was established, and the healthy hemisphere could gain control over the paralyzed arm (Hua et al., 2015). As reported in our recent trial, CC7 surgery in combination with rehabilitation led to an average 17.7-point increase in the Fugl-Meyer score, significantly exceeding the magnitude of improvement in the control group, who underwent rehabilitation only (Zheng et al., 2018a). In addition to improvements in arm motor function, spasticity and difficulties performing activities of daily living were also ameliorated (Zheng et al., 2018a). CC7 surgery is an entirely novel approach for stroke recovery, and more effort should be made to promote CC7. Considering the essential roles of nursing in all medical treatments, establishing a standard perioperative nursing program may be a promising approach.

The nursing program was mainly developed on the basis of the following two aspects: patient characteristics and CC7-related adverse effects. The former aspect mainly includes advanced age and many comorbidities, which require detailed preoperative assessments and education (Writing Group M et al., 2016). Moreover, because most stroke survivors have difficulties performing activities of daily living, the nursing program also focuses on providing assistance in daily activities and preventing accidental injuries (such as falls) (Quigley, 2016). The injuries include hematoma and injuries of surrounding nerves (e.g., the superior laryngeal nerve, the recurrent laryngeal nerve and the phrenic nerve), which may lead to hoarseness and dyspnea. Additionally, paresthesia is usually reported after nerve transfer (Wang et al., 2018), and the management of paresthesia, especially in the presence of severe pain, is also a topic of concern. Targeted care for the above adverse effects is essential for recovery.

Accordingly, this paper proposes a perioperative nursing plan for CC7: the Huashan nursing program. The key aspects of nursing work were addressed to ensure patient safety and successful surgery and reduce adverse events. Moreover, we performed descriptive statistical analyses of the clinical data of 85 patients admitted to our center to demonstrate the clinical outcomes of the Huashan nursing program intervention.

## Methods

### Participants

A total of 85 participants were included in the study, and they underwent CC7 (Xu, 2020; Jiang et al., 2019; Zheng et al., 2018b) surgery at Huashan Hospital from June 2015 to June 2018. The inclusion and exclusion criteria were similar to those in our previous study (Zheng et al., 2018a).

### The Huashan nursing program

**Fig. 1:**
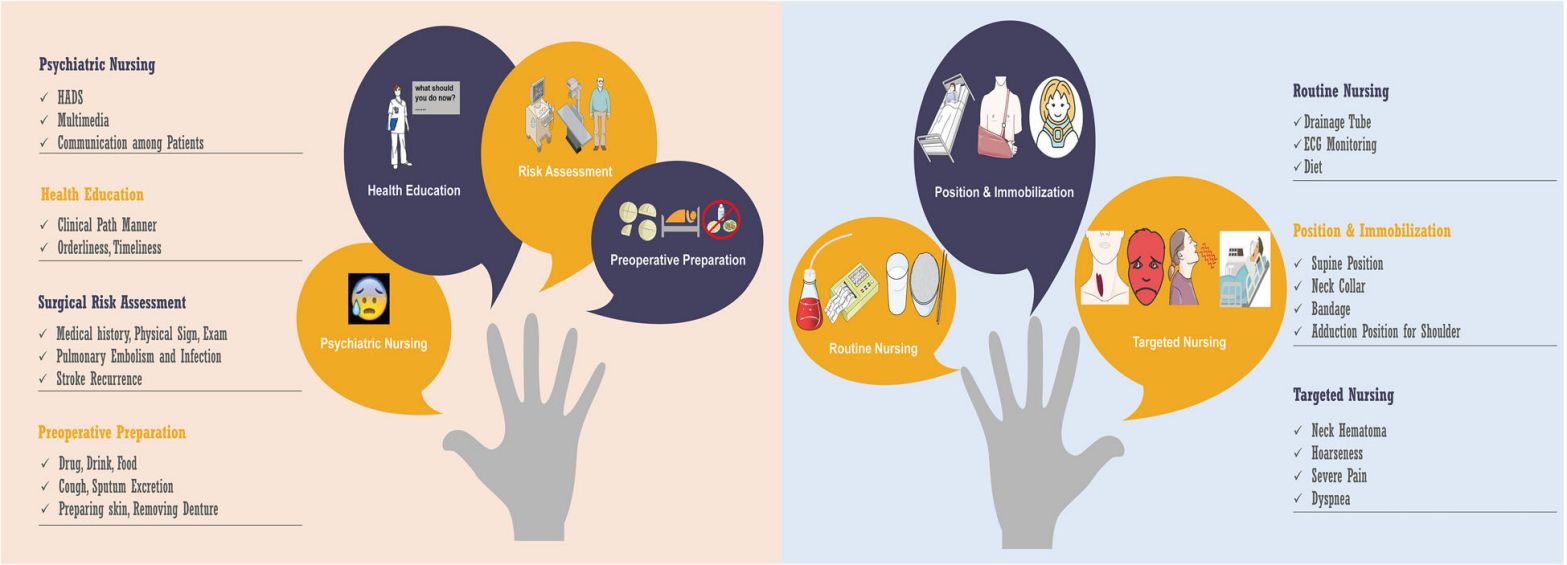
Illustration of the Huashan nursing program for stroke patients undergoing contralateral seventh cervical nerve transfer. Preoperative care mainly included psychiatric nursing, health education, risk control, and general preoperative preparation, while postoperative nursing mainly consisted of routine nursing, patients’ position and immobilization and targeted nursing for adverse events

The Huashan program is illustrated in Fig. 1, and the details are elaborated as follows (Levett et al., 2016; Roulin et al., 2017):

A. Preoperative nursing

Psychiatric nursing: All patients were educated after admission to the hospital, mainly on CC7-related principles, possible complications, and reasonable postoperative expectations. In addition, the hospital anxiety and depression scale (HADS) was used to assess the psychological states of the patients, and special attention was given to the patients with a score ≥ 8 (Snaith, 2003). We encouraged communication among patients to eliminate negative emotions and build patients' confidence in treatment, and multimedia materials were also used to share information on CC7.

Clinical path in health education: We outlined the entire plan of care a patient is expected to follow throughout the treatment course. Both orderliness and timeliness were emphasized, and education was scheduled in care pathway (Liu et al., 2014). We taught the patients how to cooperate with the preoperative preparation, the purpose of each preoperative examination, and the corresponding management of abnormalities (details seen in the “Surgical risk assessment” and “Preoperative preparation” sections). We also informed the adverse effects that may be encountered after surgery and the corresponding countermeasures. The pattern of the peripheral nerve regeneration and brain plasticity after surgery, the expected functional recovery, and the basic principles of postoperative rehabilitation will also be presented (Li et al., 2020). This path will lead to a better understanding and cooperation of patients and promote efficient and orderly operations for nursing work.

Surgical risk assessment: This assessment is essential, as most stroke survivors are characterized by an advanced age and many comorbidities (Writing Group M et al., 2016). Special efforts are made to prevent pulmonary embolism, pulmonary infection, and stroke recurrence. Therefore, it is essential to determine whether the patients have deep vein thrombosis, whether the severe stenosis or unstable plaque exists in the head arteries, whether there is severe decline of lung function. Details regarding the preoperative examinations performed are listed in Table 1. The patient’s medical history and physical signs are of great significance, and examinations should be performed appropriately to minimize risk.

**Table 1:** Pre-CC7 examinations

	Examinations	Examination purpose
**Basic examination**	Routine blood analysis, coagulation function test, electrocardiogram, tests of viral hepatitis, syphilis, and HIV	Routine preoperative assessment
**Specific examination**	Echocardiography	Identify cardiopulmonary function is acceptable for surgery Identify mural thrombus is absent
	Pulmonary function test	
	Carotid artery ultrasound	Identify that there is no severe vascular stenosis or unstable plaque
	Head CT angiography	
	Lower limb deep vein ultrasound	Identify no deep vein thrombosis exists
	Brachial plexus MRI	Identify that there is no anatomic variation
	Brain functional MRI	Brain plasticity assessment

Preoperative preparation: Routine preparation included cessation of antiplatelet and anticoagulant drugs 5 days before surgery; fasting (water and food) for 6 h before surgery; removing the denture; training for cough and sputum excretion; and preparing the skin of both axillae, the jaw and the uninjured limb (for the preparation of a sural nerve graft).

B. Routine postoperative nursing

The patients were positioned supine after the surgery. Two negative-pressure drainage tubes were used, and the color, quality, and quantity of drainage fluid were monitored. Generally, the drainage tubes were removed when the color of the drainage fluid turned faint yellow, and the volume collected over 24 h was less than 20–30 ml. When the volume increased suddenly or the color turned bright red, we checked for active bleeding and reported it in a timely manner. In contrast, when the volume decreased suddenly, an obstruction could have occurred, and we needed to identify and resolve the problem. Meanwhile, sandbags were placed over the bilateral clavicle for 48 h to reduce bleeding and the incidence of hematoma. The patient’s state, particularly regarding complaints of chest tightness, difficulty breathing, and neck swelling, was closely monitored.

ECG monitoring and oxygen inhalation were performed for 4–6 h after surgery. Blood pressure management is essential for the prevention of cerebrovascular accidents. We instructed the patients with hypertension to continue antihypertensive therapy as usual. Low ambient temperature, anxiety, pain, and sleep disturbances can increase blood pressure, and the impact of these factors should be minimized as much as possible (details are provided in the next section).

Esophageal edema may occur due to the stimulation or pulling of the esophagus during the operation; thus, postoperative dietary guidance is required to protect the esophagus. A liquid diet was allowed 6 h after surgery, and a semiliquid diet was provided on postoperative day 2. From postoperative day 3 onward, the patients consumed a soft diet, and after 1 month, the patients consumed a general diet. For the prevention of lung infection, early out-of-bed activity was encouraged, and the range and intensity of activity was gradually increased. For the prevention of lower extremity deep venous thrombosis, an intermittent pneumatic compression device was utilized.

III. Positioning and immobilization

To reduce the magnitude of traction on the transferred C7 nerve, a neck collar and bandage were used to reduce the movement of the head and paralyzed arm, respectively, for 4 weeks after surgery (Li et al., 2020). The shoulder on the paralyzed side was maintained in an adducted position, and we helped the patients hold a towel with the paralyzed hand to relieve spasticity.

IV. Targeted nursing for adverse events

**Fig. 2:**
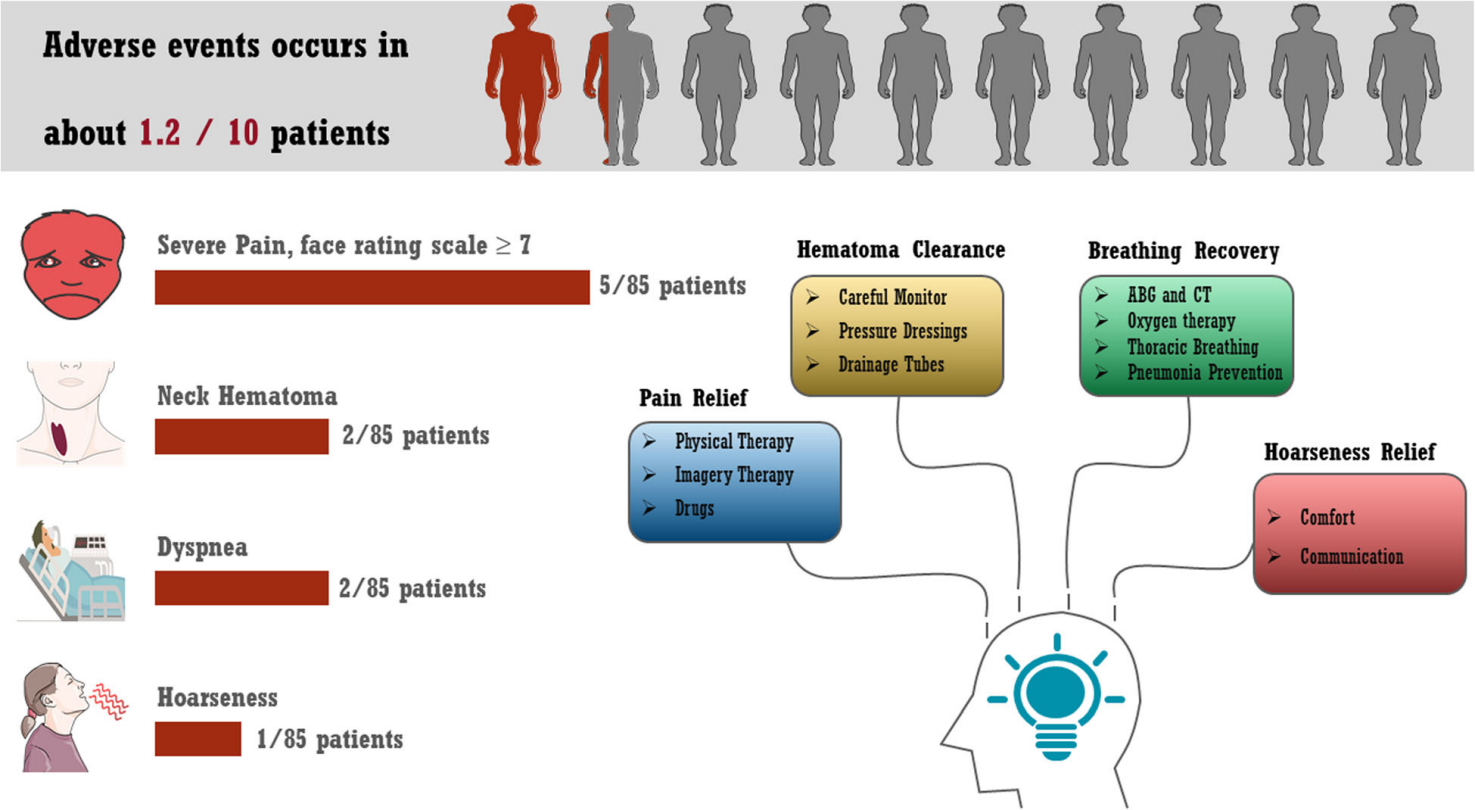
CC7-related adverse events and the targeted nursing program. The occurrence and manifestation of postoperative adverse events are illustrated. A targeted nursing program was proposed for pain relief, hematoma clearance, breathing recovery and hoarseness recovery

As shown in Table 2, adverse events occurred in 10 patients after surgery. Targeted nursing for the above adverse events played essential roles in recovery, and the details are as follows (Fig. 2).

**Table 2:** Demographic data and adverse events

		No. (%)
Sex	Male	74 (87.06%)
	Female	11 (12.94%)
Side of paralyzed hand	Left	45 (52.94%)
	Right	40 (47.06%)
Cause of injury	Cerebral hemorrhage	48 (56.47%)
	Cerebral infarction	37 (43.53%)

Neck hematoma. Attention was given to the self-reported symptoms of patients and the quality and quantity of the drainage liquid. When the amount of drainage liquid increased and the color turned dark, we needed to remove the neck collar, watch the color of the neck, observe whether the neck widened, and palpate the neck to assess muscle tension. Neck hematoma is usually caused by poor drainage and the use of anticoagulant drugs. For obstructed drainage, the tubes were repositioned. For the patients with a medical history of anticoagulant drugs, pressure dressings were effective, and drainage tubes were not removed unless the situation improved. It should be noted that some hematomas cannot be relieved by the above methods. In this case, a timely notification to the doctor is necessary. After comprehensive assessments, other approaches could be used, such as aspiration.

Hoarseness. Injuries of the recurrent laryngeal nerve can lead to hoarseness, which is generally caused by excessive traction during surgery or neck hematoma compression (Myssiorek, 2004). Monitoring of changes in voice or coughing when drinking should be performed, especially in patients with neck hematoma. For patients suffering from nerve traction, hoarseness spontaneously resolves within approximately 1 week, and doctor-patient communications were increased to relieve patients’ anxiety. Regarding patients with neck hematoma, treatments of the primary disease were emphasized, and the details are listed above. For nerve traction, hoarseness spontaneously resolves, but attention was given to patient comfort and communication with nurses to facilitate a positive mood in patients.

Severe pain. Pain and numbness of the arm were the most common adverse events after surgery (Zheng et al., 2018a). In severe cases, sleep disorders, anxiety and depression, and fluctuations in blood pressure and blood glucose levels can occur, which ultimately negatively impact recovery. The faces pain scale (FRS) was utilized to assess pain severity (Hicks et al., 2001). For patients with a score < 5, the preferred treatment was physical therapy. For patients with a score between 5 and 7, we guided imagery therapy, and the patients were asked to relax their minds and imagine positive scenarios, which attenuated sympathetic activation to relieve pain. For patients with a score higher than 7, sleep disorders can occur, and drug therapy is often necessary. Painkillers (i.e., gabapentin and pregabalin) and sleeping pills were used as needed.

Dyspnea. Phrenic nerve injury can lead to dyspnea (Kokatnur & Rudrappa, 2018), which is usually caused by excessive traction or accidental injury when the scalenus is cut anteriorly. We closely monitored the patients’ self-reported symptoms, respiratory frequency, and oxygen saturation. When dyspnea occurred, a high concentration of oxygen was used, and emergent blood gas analysis and chest computed tomography (CT) scans were performed to determine the severity. Afterward, patients were asked to enhance thoracic breathing. Exercise was performed three times on the first day for 10 min each time. Then, the duration and intensity of breathing exercises improved gradually. To prevent lung infection, the head of the bed was raised to 30°, the patient was assisted in turning over, the back of the patient was patted, and atomization inhalation was performed (Gohl et al., 2016; Takemura, 2020).

### Data analysis

Descriptive statistical analyses were performed for the demographic data and adverse events. Regarding CC7-related adverse events, we reported the incidence and severity of the events and the outcomes after treatment.

### Patients’ characteristics

The mean patient age was 53.2 years, and the SD was 7.4 years. Furthermore, the sex, affected side, cause of injury, and occurrence of adverse events are shown in Table 2.

### Adverse effects and targeted nursing

A. Clearance of neck hematoma

Two patients (2.35%) showed neck hematoma after surgery, both of whom had a medication history of warfarin. The drainage liquid exceeded 200 ml within 24 h after surgery, and the neck turned cyanotic and widened. Under such conditions, the patient’s vital signs should be monitored carefully, especially oxygen saturation. Meanwhile, pressure dressings were utilized, and the drainage tubes were left in place until the situation improved. After treatment, the hematoma was cleared, and the drainage tubes were successfully removed in both patients.

B. Relief of hoarseness

One patient (1.18%) exhibited hoarseness due to traction of the recurrent laryngeal nerve. Comfort and communication with nurses were essential in ensuring that the patient’s mood remained stable, which contributed to complete symptom resolution.

III. Pain relief

To date, five patients (5.88%) exhibited severe pain (i.e., FRS score > 7) as well as sleep disorders after surgery. A treatment program involving communication and comfort, physical therapy, imagery therapy, and drug therapy was performed, and the FRS score gradually decreased to less than 2 points in all five patients.

IV. Breathing recovery

Two patients (2.35%) reported dyspnea on postoperative days 1 and 2, and the oxygen saturation had decreased to 92–94%. Oxygen therapy, emergent blood gas analysis, and chest CT scans were performed immediately. Moreover, thoracic breathing was encouraged, and preventive measures for pneumonia were also emphasized. After the above treatments, both patients achieved normal breathing, and the oxygen saturation rate remained above 97%.

## Discussion

We proposed a perioperative nursing program, the Huashan program, for stroke patients undergoing CC7. Furthermore, a preliminary study enrolling 85 patients suggested its feasibility and effectiveness.

Before surgery, nurses focused on performing a comprehensive examination. As mentioned earlier, stroke patients undergoing CC7 surgery are generally old and have various comorbidities, such as severe cerebral artery stenosis, venous thrombosis, and cardiopulmonary disability, which will greatly increase the risk of surgery. A series of standardized and comprehensive preoperative examinations helped to exclude patients with contraindications and represented an essential step for ensuring surgical safety. Moreover, during the health education process, the nurse told the patients of the steps they expected to go through during the entire treatment process and reasonable expectations for functional improvement. The education contributed to reduce patients’ worries and build their confidence (Hofmann, 1993; King et al., 2016). Regarding postsurgical nursing, attention should be given to the management of surgery-related complications, especially neck hematoma, hoarseness, severe pain, and dyspnea (Zheng et al., 2018a). Nurses needed to pay close attention to the patient’s main complaint and clinical manifestations, such as whether there was abnormality in the drainage fluid, whether the swelling occurred in the neck, whether the patient’s voice changed, etc. Once the above phenomenon happened, the nurse should report to the doctors and performed the symptomatic treatment in time. In addition to the early discovery and timely treatment of complications, the prevention of accidents (for example, fall prevention) and treatment of comorbidities (for example, the treatment of hypertension and diabetes) by nurses must also be emphasized.

There were several methodological issues in this study. The sample size is relatively small. The article would be much stronger if it were able to compare participants in control and intervention group. Further study with larger sample size and a randomized controlled design will help to confirm the efficiency of Huashan nursing program.

To conclude, CC7 is a novel and effective treatment approach for patients with spastic arm paralysis in the chronic stage. Based on the patients’ characteristics and surgery-related adverse effects, the present program provides a feasible template. The Huashan nursing program helps maximize the benefits of CC7. Moreover, it can also provide novel perspectives of nursing for patients with brain injury and patients undergoing nerve transfer.

## Data Availability

The datasets used and/or analyzed during the current study are available from the corresponding author on reasonable request.

## Abbreviations

CC7: Contralateral seventh cervical nerve transfer
HADS: Hospital Anxiety and Depression Scale
FRS: Faces pain scale
